# High-level talents’ perceive overqualification and withdrawal behavior: A power perspective based on survival needs

**DOI:** 10.3389/fpsyg.2022.921627

**Published:** 2022-11-03

**Authors:** Caiyun Huang, Siyu Tian, Rui Wang, Xue Wang

**Affiliations:** ^1^College of International Economics and Trade, Ningbo University of Finance and Economics, Ningbo, China; ^2^Ningbo Philosophy and Social Science Key Research Base “Regional Open Cooperation and Free Trade Zone Research Base”, Ningbo, China; ^3^College of Business, Shanghai University of Finance and Economics, Shanghai, China; ^4^College of Business, Wenzhou University, Wenzhou, China

**Keywords:** perceived overqualification, withdrawal behavior, the sense of power, protected values, feeling trusted

## Abstract

Based on the power basis theory, this study examined the relationship between high-level talents’ perceived overqualification (POQ) and withdrawal behavior and the mediating role of sense of power. We also analyze the boundary effects of protected values and being trusted. The hypotheses of this study were tested through questionnaires gathered across three phases over 3 months from 371 high-level talents from 6 enterprises, 5 governments, and 13 universities in China. Hierarchical regression analyses and bootstrapping appraisals showed that: (1) POQ has a positive relationship with withdrawal behavior; (2) sense of power mediates the relationship between overqualification and withdrawal behavior, with high POQ resulting in low perceived power, which then reinforces withdrawal behavior; (3) the negative relationship between POQ and sense of power is stronger for high-level talents with high protected value (as opposed to low); (4) the negative relationship between sense of power and withdrawal behavior is stronger for high-level talents with highly being trusted (as opposed to low); (5) moderated-mediation analyses reveal that the mediating effects of sense of power are stronger for employees with high (as opposed to low) protected values or being trusted. At the end of this study, the theoretical and practical implications are discussed.

## Introduction

Following COVID-19 and the increase in unemployment, society has a greater need for talents, and an increase in the number of highly gifted individuals who find themselves in less-than-ideal employment situations (Alosani and Al-Dhaafri, 2021). The phenomenon of overqualification (i.e., people who have more education, skills, and knowledge than are required for their jobs; Maynard, 1998) has drawn increasing attention (Zhang et al., 2020). According to a growing body of research, overqualification can have negative consequences on workers and organizations, including significant losses in personnel, resources, and cash (Arvan et al., 2019). With the implementation of China’s “Ten Thousand Talents Plan,” many localities have launched a series of policies to introduce and reward high-level talents. Under this background, more and more high-level talents perceive overqualification (Maynard, 1998) and frequently flow. In China, the explanations of those flow have placed more emphasis on objective factors like policy, economic development, legislation, and the market (Chen et al., 2018), instead of focusing on subjective issues like POQ. It is vital to investigate the relationship between high-level talents’ POQ and withdrawal behaviors.

Overqualification refers to the skills, knowledge, and abilities possessed by individuals who exceed the needs of the post. The two types of overqualification are objective overqualification and subjective overqualification. The former speaks of a discrepancy between a candidate’s skills and the credentials necessary for the post. The latter refers to the subjective view of this mismatch, also known as the perceived overqualification (POQ; Khan and Morrow, 1991), which refers to the perception that individuals are equipped with excessive qualifications for a job (Zhang et al., 2021). POQ will have a stronger impact on employees’ attitudes and behavior even though objective overqualification can more accurately represent the facts (Arvan et al., 2019). As a result, this study concentrates on the POQ of high-level abilities and explores its implications.

Currently, research on the outcome variables of overqualification mainly focuses on active behavior and creativity, such as job satisfaction, organizational commitment, wellbeing, innovative behavior, organizational citizenship behavior, job involvement, and performance (Harari et al., 2017), but little attention is paid to unproductive behavior. The anti-productive behaviors caused by overqualification are mainly those that will not cause too much harm to the organization (Fine and Edward, 2017), such as withdrawal behavior (Harari et al., 2017; Chen et al., 2020). Even though some researchers explored the relationship between POQ and withdrawal behavior (Harari et al., 2017; Chen et al., 2020), little focus is on the satisfaction of needs, which is an important condition for individual survival and development. Hence, the central aim of this study is to expand upon recent investigations of the relationship between high-level talents’ POQ and their withdrawal behavior from a new perspective of the satisfaction of needs.

Based on the power basis theory (Pratto et al., 2011), this study proposed that POQ influences withdrawal behavior mainly through a sense of power. According to the theory, power is the capacity of individuals or groups to meet their own basic demands for survival (Pratto, 2016), and people must adapt to environmental constraints and supplies to achieve their survival goals (Pratto et al., 2011). Three variables affect whether these demands are met: the skills of individuals or groups, the environment, and the ability of the environment to fully utilize the skills of individuals or groups (Pratto et al., 2008). Individual power increases when people can fully utilize their talents (low POQ). On the contrary, when people are unable to fully utilize their talents (high POQ), their power is diminished, and they find other ways to meet their demands. The typical way is to transfer the “battlefield,” that is, retreat behavior (Pratto, 2016).

To understand why an employee who perceives overqualification is likely to behave in withdrawal behaviors, we need to examine the mechanism of the sense of power. Meanwhile, an equally crucial issue is what factors will affect the effects of POQ. Additionally, this study examined how the protected values of employees and being trusted by supervisors qualify their reactions to POQ. The level of education is one of the key indicators for identifying high talents and abilities, and education’s key goal is to help those who have it develop strong moral principles. Therefore, high-level talents have more obvious protected values, such as the absolute value or principle of refraining from engaging in any commercial transactions (Baron and Spranca, 1997). When employees have POQ, they are unwilling to meet their needs through other means, thus reducing the impact of POQ on power. In addition, high-level talents have a stronger ability to obtain resources, which makes them a greater probability of being trusted. Trust can bring organizational commitment and pressure, which can alleviate the withdrawal behavior caused by the decline of power. This study argues that if employees feel trusted and have strong protected values, their POQ will increase their sense of power, and further change their withdrawal behaviors.

This study makes several theoretical contributions. First, this study focuses on the POQ of high-level talent and provides a new explanation for high-level talent attrition. Previous research on POQ had a lesser emphasis on high-level talents, and discussed external factors for the brain drain without considering individual factors at the psychological level (Chen et al., 2018). Sequentially, we argued that high-level brain drain should take into account their inner feelings in addition to policy, legal, and market factors. High-level talents are very mobile due to the simultaneous pressures of the knowledge economy and COVID-19, which makes it easy for them to choose jobs that do not match their skills and results in POQ. As a result, investigating the link between POQ and withdrawal behavior can offer a more in-depth explanation of the nature of talent loss as well as more specific practice advice. Therefore, exploring the relationship between POQ and withdrawal behavior can provide a more microscopic explanation of the nature of talent loss as well as more detailed guidance from practice.

Second, our research explored the relationship between POQ and withdrawal behavior, which could enrich the research between POQ and counterproductive behavior. Relatively little research has been conducted on counterproductive behaviors, while some studies discussed the effects of POQ on proactive behaviors (Harari et al., 2017). Although these counterproductive behaviors are not particularly damaging to the organization, they can eventually lead to a continuum of behaviors and encourage staff to engage in aggressive and detrimental behaviors (Seitz et al., 2000). Investigating the relationship between POQ and counterproductive behaviors is therefore required. In addition, previous research, which mostly concentrated on a single domain, lacked a direct examination of the link between POQ and withdrawal behavior (e.g., Maynard and Parfyonova, 2013; Harari et al., 2017). We investigate the impact of POQ on withdrawal behavior in high-level talent, which is a useful addition to previous studies.

Third, we discussed the mediating effect of the sense of power from the perspective of survival needs satisfaction with the help of the power basis theory. This theory provides a critical viewpoint on the mechanism of POQ. Existing POQ studies have analyzed from the perspective of social exchange, resources, and equity (e.g., Fine and Edward, 2017; Chen et al., 2020), and have not discussed the satisfaction of basic human needs. On the other hand, psychological needs and motivation are the primary forces that influence a person’s behavior (Sheldon et al., 2001), and they can be interpreted as the internal nature of POQ. When individuals develop POQ, their requirements for material resources, physical and psychological integrity, competent environmental interaction, care, belonging, and reproduction are not satisfied. A decrease in one’s sense of power, coercive power, resource control power, knowledge power, relational power, juridical power, and self-transcendence power results, which encourages people to seek out needs-satisfying activities from other organizations, which is known as withdrawal behavior. To address the core issue of why high-level talents depart when POQ arises, this study draws on the underlying power foundation theory. The study explains the issue in terms of the satisfaction of survival needs.

Fourth, this study explores the boundary role of employees’ protective values and being trusted by supervision from both individual and leader perspectives, suggesting new boundary conditions for the relationship between POQ and withdrawal behavior. A few studies have analyzed at the individual level (e.g., Ma et al., 2020; Schreurs et al., 2020; Woo, 2020), considering individual characteristics. We combined factors at the individual and leadership levels, primarily considering how they shifted their focus from self to others. High-level talent has a greater level of protected values and feeling trusted by supervisors as a result of their education and competencies. As a result, the moderating variables selected for our study highlight the unique characteristics of high-level talent. To comprehend the connections between POQ and withdrawal behavior, we also provided a contingency perspective. The discussion of boundary conditions delineated clearer conditions for the main effect of POQ.

## Theory and hypothesis

### Power basis theory

The power basis theory explored the reason why people need power, and it always appears individual life, and also explained the social inequality that is the by-product of power studies (Pratto et al., 2011). This theory suggests that the existence of power is that humans must use power to survive and thrive in the context of environmental constraints and affordances (Pratto et al., 2011). Therefore, defining power as a “right of control” or “right of action” is not complete. Power is the capacity to satisfy individuals’ or groups’ demands. In our study, the term “needs” refers to necessities of life, encompassing 6 cases: the need for physical and mental wholeness—constructive power; the need to use resources—control of resources power; the need to interact skillfully with one’s surroundings—knowledge power; the need for other people’s care—relationship power; the need for social approval—legitimacy power; and the need for reproduction—self-expansion power.

The action to meet the needs and the degree to meet the needs depends on the individual or group’s ability, the ecological environment, and whether the environmental supply is suitable for the ability to play (Pratto et al., 2008). Especially, “environment” refers to both the physical and social aspects of existence, including lifestyle and interpersonal relationship. In the event of sufficient supply and appropriate development, high-level talents could also possess high-level power, and vice versa. If the environment cannot accommodate their skills’ exertions, that is, the need cannot be met, they would switch to a different environment. Narayanan et al. (2013) found that high-power individuals prefer to seek new social connections to satisfy their demands and find social inclusion after being socially excluded. The intensity of power pursuit and perceived power are further influenced by the sensitivity to requirements If employees are more sensitive to need satisfaction, they will be influenced more to seek new opportunities when the environment is not suitable for their performance.

### Perceived overqualification and withdrawal behavior

POQ means that employees first advance their knowledge, skills, and talents, then adapt those skills to the demands of the job, leading to the sense of a mismatch between personal traits and job demands (Maynard and Parfyonova, 2013). These employees believe they deserve better treatment than they currently receive, which frustrates them and causes them to act in unfavorable ways (Liu and Wang, 2012), such as having unfavorable attitudes at work, feeling unwell, or acting in an unproductive manner (Luksyte et al., 2011; Liu et al., 2015; Harari et al., 2017). In line with the power basis theory, employees would not quit if their demands for survival were addressed, however, if these survival needs are not met, they will find other ways to survive (Pratto et al., 2011). Taken as a whole, employees will generate withdrawal behaviors when they are in POQ.

Laziness, leaving work early or on time, dismission, job searching, and retirement are all examples of withdrawal behavior, which is a group of actions taken by workers who leave their jobs or perform non-performance work duties (Hanisch and Hulin, 1990). The withdrawal behavior is defined by poor involvement and high slack, which has a significant detrimental influence on effectiveness, particularly on productivity, management costs, and interpersonal relationships (Smith et al., 2016). It is significant to explore its antecedent variables to help reduce withdrawal behavior. Previous research on the causes of withdrawal behavior from the individual and organizational perspectives, in particular, the individual perspective focuses primarily on individual-job matching, and the organizational perspective includes leadership, organizational atmosphere (safety, effective communication, fairness, etc.), salary incentive system, task allocation, work pressure (Tak, 2011; Abubakar et al., 2017; Zhou et al., 2018). The primary cause is the poor utilization of individual resources, and they choose to daydream and slack off at work before trying to recoup resources by self-control or even acting against the interests of the company (Seitz et al., 2000).

The power basis theory showed that the desire for survival is one of the most important sources of human needs. In order to meet this need, people may create other different needs, which govern their behavior (Pratto et al., 2011). Employees who feel overqualified believe their knowledge, talents, and abilities cannot be used to their full potential within the organization, which causes them to feel as though they cannot have enough to survive, so they adjust their behaviors to meet their needs (Pratto et al., 2011). Compared with other regulating behaviors, withdrawal behavior is the preferred option (Fine and Edward, 2017), because they cost less on the withdrawal behavior. In addition, withdrawal behavior can help individuals to spend more time on activities outside work to regain satisfaction (Bakker et al., 2003). In addition, if working requirements are insufficient for an employee’s skill level, the person will generate a sense of boredom or loss, feel useless and bored, and even lose interest in work, resulting in withdrawal behavior (Bakker et al., 2003). Studies have shown that POQ can cause a range of withdrawal behaviors, including the desire to resign, the intention to look for a new job, time encroachment, and unproductive conduct (Maynard and Parfyonova, 2013; Harari et al., 2017; Chen et al., 2020; Schreurs et al., 2020). Therefore, we proposed the following hypothesis:

Hypothesis 1: POQ *is positively related to* withdrawal behavior.

### The mediating effect of the sense of power in the relationship between perceived overqualification and withdrawal behavior

As one of the core structures of human society (Russell, 1938), power is the important motivation for behaviors that include workplace behaviors (Belmi and Pfeffer, 2016). It is known as “the main organizing force of social life” (Van Kleef et al., 2011). To profoundly explain the origin and dynamics of power, the power basis theory defines it as the ability to meet the needs of survival (Sun and Pratto, 2017). This theory suggests that the factors that affect the satisfaction of individual survival needs are their own talents, environment, and the extent to applying talent (Pratto et al., 2008). The definition of power states that a person can act in accordance with his or her own desires (Weber, 1947). That is to say, a powerful person can pay attention to their immediate demands and objectives (Guinote, 2007). Hence, the capacity to meet requirements symbolizes power. If the environment could produce talent, the employee would be more likely to meet their demands, leading to the development of a strong sense of power, and vice versa.

The environment of the organization is suitable for survival, or the organization can provide them with a variety of support conditions, such as greater funds, convenient conditions, the care of leaders, and learning opportunities. These can give full play to their talents, that is “birds fly when the sky is high, fish leap when the sea is wide.” It is simpler for them to use their skill to satisfy demands like being respected and acknowledged by colleagues, gaining deeper relationships with colleagues, obtaining more resources, and influencing the satisfaction of others’ needs. On the contrary, if the organization’s resources are insufficient, high-level talents will not be able to work since they will be hindered or lack the necessary resources. To put it another way, even a skilled housewife cannot prepare dinner without rice. As a result, the requirements for high-level skills will not be satisfied, and the associated power will be diminished (Pratto et al., 2011; Sun and Pratto, 2017). Referring to the definition of POQ, the organizational environment is insufficient or not suitable to develop the ability, which causes a barrier to satiate one’s survival demands and a weakening of power. Because of this, POQ makes people feel less powerful.

In light of the power basis theory, individuals are unable to satisfy their survival needs when the sense of power is low, so they will try to switch to seek satisfaction from others (Pratto et al., 2011). Furthermore, the approach/inhibition theory of power (Keltner et al., 2003) proposed that high power generates approach tendency, and low power generates inhibition tendency, that is, low power prefers to give rise to withdrawal behavior. Low power also influences withdrawal behavior through various negative emotions, such as anxiety, embarrassment, fear, sadness, and guilt (Keltner et al., 2003). Berdahl and Martorana (2006) discussed poverty in the United States and the results found that low-power subjects experienced more negative emotions. Furthermore, individuals will generate negative emotions when they have a low level of power triggered by high POQ. The negative emotions come into withdrawal behavior (Liu et al., 2019). Liu et al. (2019) showed that negative emotions are the mediated effect of the relationship between identity difference and withdrawal behavior. Hence, the unsatisfaction of survival needs generated by POQ will make individuals have a low sense of power, and then make individuals withdraw from work to seek new opportunities.

Specifically, we hypothesize the following:

Hypothesis 2: The sense of power is the mediated effect of the relationship between POQ and withdrawal behavior.

### The moderating effect of protected values

Protected values are absolute, non-negotiable values and moral principles, and they cannot be traded economically with other values (Baron and Spranca, 1997; Pratto et al., 2011). For example, the fact that things like life, health, nature, love, honor, justice, and human rights are sacred demonstrates a super-reality. And they are secular values, which can never be traded with money. In practice, these forms of values date back to ancient times. As the quote, we will never alter our original aspiration because of riches, poverty, or force. Many memorial persons, like austerity, martyrdom, and missionaries, might be hyped out with this faith. In addition to assessing people’s interests when influencing their decisions, protected values also serve as a major source of inspiration for people throughout their lives. The study makes two claims as to why protected values are effective. First, it claims that protected values are constructed and represented according to an absolute moralism rule, which allows people to make decisions when faced with material temptations and threats without considering pros and cons and instead rely solely on the rules and values that they were raised with. Second, protected values have a high-value significance since they are related to self and moral self-identified. Once individuals are forced to choose between values and money, their moral identity will be threatened and negative emotions of anger and disgust will arise to counteract the temptation of money and reinforce the original values (Pratto et al., 2011). Pretus et al. (2019) studied immigrants with extremely religious protected values and found that these individuals were extremely committed to religious values, even willing to die for them and that the trade-off between pros and cons did not play a role at all in their entire brain activity.

Employees will have a poor sense of power if they believe they are overqualified and the organizational environment is unsuitable for fostering their skills. As a result, their survival needs won’t be satisfied. Employees with strong protective values will adopt the mindset that breaking jade is preferable to breaking tile, and they won’t compromise their morals and values to fit the organizational culture or engage in financial transactions to suit their survival needs (Pratto et al., 2011). In contrast, employees with low protected values have a higher probability of integrating into the organization through economic transactions to meet survival needs. That is, the highly protected values will have a significant detrimental effect on POQ’s perception of power. What’s more, individuals with highly protected values exhibit strong negative emotions in the event of a forced change in their values. Duc et al. (2013) found that theology majors activated more brain area activity associated with anger and disgust in the face of forced choices for life and monetary compensation. This demonstrates that those who have strong values that are safeguarded are more vulnerable to the possibility of value violation (Pratto et al., 2011). These employees experience POQ and believe that their talents are not treated fairly (Bakker et al., 2003), which causes them to react more strongly when their protected values are threatened. As a result, the organization should emphasize the negative effects of POQ on perceptions of power. This study hypothesized that:

Hypothesis 3a: Protected values negatively moderated the relationship between POQ and the sense of power. The negative relationship between POQ and the sense of power could be strengthened when the level of protective values was high, and vice versa.

This study further proposes that protected values moderate the mediating role of the sense of power between POQ and withdrawal behavior. Employee withdrawal behavior will be influenced by POQ through the sense of personal authority if employees feel strongly about their protected values. When the level of protected values is low, POQ will influence withdrawal behavior less through the sense of personal power. Therefore, the mediating hypothesis being moderated is proposed.

Hypothesis 3b: Protected values moderate the mediating sense of power between POQ and withdrawal behavior. The mediating role of the sense of power is enhanced when the level of protected values is high and diminished when the level of protected values is low.

### The moderating effect of the feeling of trust

Trust is the ability to anticipate the best in people despite their flaws (Song et al., 2019). Employees who trust their leaders, leaders who trust their employees, and employees being trusted by the leader are the three key areas where research on trust between leaders and employees has been done. Compared to the other two topics, being trusted by supervisors has received much less research. In recent years, researchers have found that being trusted by supervisors, if insufficient, has a greater impact on the trust relationship between leaders and employees and affects trust outcomes. Conversely, if employees feel enough trusted by the leader, they will have high levels of self-confidence and self-esteem, leading to good performance (Song et al., 2019). However, feeling trusted by supervisors does not always lead to positive effects, Baer et al. (2015) showed that being trusted by supervisors not only makes employees proud but also makes them feel more burdened and more concerned about upholding their reputation, which increases emotional exhaustion and lowers performance. Thus, the effect of being trusted by supervisors on job performance is a double-edged sword.

When subordinates feel that their leaders trust them, it is because the leaders have given them power, treated them fairly, offered support, or shared crucial information with them (Deng et al., 2010). Although there are both positive and negative effects of being trusted by supervisors, both result in lower levels of withdrawal behaviors among high-power individuals. This is because, on the one hand, when individuals have a higher trust from supervisors, they establish a stronger psychological contract with the organization and form stronger organizational commitment (Baer et al., 2015). Due to their eagerness to repay the organization and provide additional benefits for it, employees will put out extra effort to the organization’s advantage and decrease the likelihood that they will leave (Paille et al., 2013). On the other side, feeling trusted by supervisors can have a negative impact, generating more pressure (Baer et al., 2015), this pressure reinforces the negative relationship between power and withdrawal behaviors. Drnovsek et al. (2010) found that individuals with high power are less likely to select withdrawal behaviors as an avoidance coping style and low-power individuals are more likely to choose the coping style of leaving the stressor (Drnovsek et al., 2010). Consequently, there is a stronger negative relationship between power perception and withdrawal behavior the higher the feeling of supervisory trust. The following prediction is made:

Hypothesis 4a: Feeling trusted by supervisors negatively moderated the relationship between the sense of power and withdrawal behaviors. When the level of feeling trusted by supervisors was high, the negative relationship between a sense of power and withdrawal behavior increased, and vice versa.

**FIGURE 1:**
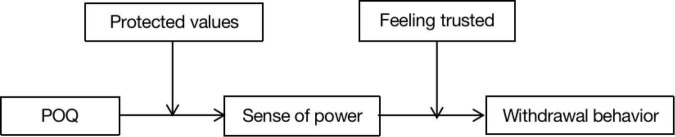
Hypothesized model.

This study further proposes that feeling trusted by supervisors moderates the mediating role of the sense of power between POQ and withdrawal behavior. Employee withdrawal behavior will be influenced by POQ through the sense of personal authority if the superiors are trustworthy. When the level of feeling trusted by supervisors is low, POQ will influence withdrawal behavior less through the sense of personal power. Therefore, the mediating hypothesis being moderated is proposed. The research model is shown in Figure 1.

Hypothesis 4b: Feeling trusted by supervisors moderates the mediating role of the sense of power between POQ and employee withdrawal behavior. The mediating role of the sense of power is strengthened when the level of feeling trusted by supervisors is high, and vice versa.

## Materials and methods

### Sample and procedures

We collected data from employees who were recruited as high-level talents and have worked for more than half a year in enterprises, universities, and government agencies, given that POQ exists in the population of high-level talent. Data were randomly collected *via* WeChat and email from 24 organizations in 6 provinces (Jiangsu, Zhejiang, Guangdong, Shanxi, Anhui, Hubei) and 2 municipalities (Beijing and Shanghai), were conducted in three waves that are separated by 3 months each. We measured POQ and protected values of employees in T1 (3rd–10th, August) by receiving 470 surveys. What’s more, we receive 415 surveys for measuring individuals’ sense of power and perception of feeling trusted by supervisors in T2 (11th–18th, September), 1 month after T1. Finally, to estimate employees’ withdrawal behavior, the study only received 371 surveys in T3 (19th–26th, October), 1 month after T2.

The sample used to test our hypotheses included 371 employees, including 43.9% female. In terms of education level, 94.6% of employees had post-graduate (master’s degree or doctoral degree). As for age, most employees in the sample are older than 28, with an amount of 289, accounting for 77.9%. In addition, we divide the employees into 3 parts in line with the working years, 22.4% of employees have worked less than 3 years, 67.7% worked for 3–10 years, and only 10% worked more than 10 years. The samples are collected mainly from enterprises and universities, with an amount of 289, accounting for 77.9%.

### Measures

The measurement inventories were translated and back-translated (Brislin and Yoshida, 1994) to assure their appropriateness in Chinese. The scales in the questionnaire were all scored on Likert 5 points, and utilized response options ranging between 1 = strongly disagree and 5 = strongly agree.

#### Perceived overqualification

At Time 1, we measure POQ using the nine-item scale by Maynard et al. (2006). Especially, the representative sample item was “The level of education required for my job is lower than my education level.” Cronbach’s alpha for the scale was 0.89.

#### Protected values

At time 1, measuring protected values used the nine-item scale by Hanselmann and Tanner (2008). And, the sample item was “I will not change my opinion no matter what the cost.” Cronbach’s alpha for the scale was 0.72.

#### Sense of power

At time 2, to estimate the sense of power, we use the eight-item scale by Anderson and Galinsky (2006), with the sample item “I could make others endorse my decisions.” Cronbach’s alpha for the scale was 0.85.

#### Feeling trusted

At time 2, the measurement of employees’ perception of feeling trusted by their supervisor adopted a 10-term scale by Gillespie (2012), with the sample item “You have the right to deal with one important matter empowered by your leader.” Cronbach’s alpha for the scale was 0.85.

#### Withdrawal behavior

At time 3, we measure withdrawal behavior using the 12-item scale by Lehman and Simpson (1992). Especially, the sample item was “I have the idea of absenteeism.” Cronbach’s alpha for the scale was 0.88.

#### Control variables

This article considered the demographic variables (gender, age, education, and total years of work) as controls due to their significant impact on the relationship between individuals’ POQ and withdrawal behavior (Abubakar et al., 2017; Harari et al., 2017; Arvan et al., 2019). Therefore, we reported the results with these controls.

## Results

### Confirmatory factor analysis

To prevent the deviation caused by the identical samples, we controlled the survey anonymously and voluntarily, separating the variables, and setting guidelines in each section. We then gathered questionnaires at three timelines. And, the results show that KMO = 0.88, Bartlett’s test of sphericity is 7402.46, *p* < 0.001. We find that it cannot precipitate any independent factors, and the variance interpretation rate of the precipitated main factor was 19.34% which was much smaller than the total explanatory variation of 59.40%, confirming the validity of the solution to the deviating problem.

We conducted a series of confirmatory factor analyses to examine the validity of the results using the factorial algorithm (Rogers and Schmitt, 2004). In this study, considering the number of questions is 48, the sample size, *N* = 371, is smaller to study. This would exacerbate the problem of the fitting index being disturbed, which can be solved by the relationship-equilibrium method. This study tested a five-factor model including POQ, protected values, sense of power, feeling trusted, and withdrawal behavior.

The item was first ranked according to weight. Next, we use a high-ratio, low-guideline to package the item into 3 or 4 observation indications. Finally, we adopt validation factor analysis by AMOS to perform the results: χ*^2^* = 230.62, *df* = 94, RMSEA = 0.06, CFI = 0.95, TLI = 0.94, SRMR = 0.06. Table 1 shows that the five-factor model provided a reasonably good fit to the data, and the other three types of models performed poorly.

**TABLE 1:** Measurement model comparisons.

Model	χ^2^	*df*	χ^2^/*df*	CFI	TLI	RMSEA	SRMR
M_0_: Hypothesized five-factor model (POQ, PV, SP, FT, and WB)	230.62***	94	2.45	0.95	0.94	0.06	0.06
M_1_: Hypothesized three-factor model (POQ&PV, SP&FT, and WB)	628.52***	101	6.22	0.81	0.77	0.12	0.11
M_2_: Hypothesized two-factor model (POQ&PV&SP&FT, and WB)	1013.79***	103	9.84	0.67	0.61	0.15	0.12
M_3_: Hypothesized single-factor model (POQ&PV&SP&FT, &WB)	1502.46***	104	14.45	0.49	0.41	0.19	0.14

*N* = 371. POQ, perceived overqualification; PV, protected values; SP, sense of power; FT, feeling trusted; and WB, withdrawal behavior; RMSEA, root mean square error of approximation; SRMR, standardized root mean square residual; TLI, Tucker–Lewis index. *p < 0.05; **p < 0.01; ***p < 0.001.

### Descriptive statistics and correlation

Descriptive statistics and correlation coefficients of our variables are displayed in Table 2. The results elaborated that POQ has a significant negative effect on withdrawal behavior (*r* = 0.22, *p* < 0.001), which verifies Hypothesis 1. However, POQ has a significant negative effect on the sense of power (*r* = −0.27, *p* < 0.001), and the sense of power has a significant negative effect on withdrawal behavior (*r* = −0.22, *p* < 0.001), which verifies Hypothesis 2, it could be further examined by hierarchical regression.

**TABLE 2:** Correlations and descriptive statistics.

Variables	Mean	SD	1	2	3	4	5	6	7	8	9
1. Gender	1.44	0.50									
2. Age	31.61	5.40	–0.03								
3. Education	2.61	0.59	−0.10*	0.30***							
4. Tenure	5.64	4.52	–0.01	0.79**	0.29***						
5. POQ	2.89	0.77	–0.06	–0.03	−0.12*	–0.03	(0.86)				
6. PV	3.38	0.51	–0.04	–0.02	0.01	0.03	0.03	(0.72)			
7. SP	3.50	0.64	−0.12*	0.13*	0.22***	0.18***	−0.27***	0.12*	(0.85)		
8. FT	3.61	0.61	−0.21***	0.15**	0.23***	0.26***	–0.01	0.24***	0.54***	(0.85)	
9. WB	2.15	0.64	0.09	−0.13*	−0.29***	−0.16**	0.22***	–0.10	−0.42***	−0.37***	(0.88)

N = 371. Coefficient alphas are in brackets. Gender was coded as follows: 1 = male, 2 = female. Education level was coded as: 1 = undergraduate 2 = master 3 = doctor. *p < 0.05; **p < 0.01; ***p < 0.001.

### Hypothesis testing

We examine mediating effects and moderating effects using hierarchical regression by SPSS, and the results are exhibited in Table 3. Hypothesis 2 predicted that the impact of POQ on employees’ withdrawal behavior is moderated by a sense of power. This can be supported by our findings. The path coefficients from POQ, to sense of power (M2: β = −0.25, *p* < 0.001), to withdrawal behavior (M5: β = 0.20, *p* < 0.001), were both statistically significant. What’s more, we incorporate the notion of the power factor into M6. It found that the coefficients for the sense of power (M6: β = −0.34, *p* < 0.001) and for POQ (M6: β = 0.12, *p* = 0.018) are significant. In addition, we adopt a bootstrap appraisal to confirm Hypothesis 2. The results show that the indirect effect of POQ on the employee’s withdrawal behavior through the sense of power was 0.08 in the confidence interval [0.05, 0.13], obviously eliminating the value of zero.

**TABLE 3:** Hierarchical regression results.

Variables	Sense of power			Withdrawal behavior					
	M1	M2	M3	M4	M5	M6	M7	M8	M9
1. Gender	−0.11*	−0.12*	−0.12*	0.06	0.08	0.04	0.01	0.04	0.02
2. Age	–0.09	–0.09	–0.06	0.04	0.04	0.01	–0.03	–0.00	–0.03
3. Education	0.18*	0.15**	0.15**	−0.26***	−0.23***	−0.19***	−0.17***	−0.18***	−0.16**
4. Tenure	0.20*	0.20*	0.17*	–0.12	–0.12	–0.06	0.03	–0.04	0.03
3. POQ		−0.25***	−0.24***		0.20***	0.12*		0.11*	0.13**
5. PV			0.09					–0.04	
6. POQ × PV			−0.10*					0.10*	
7. SP						−0.34***	−0.30***	−0.32***	−0.25***
8. FT							−0.20***		−0.22***
9. SP × FT							−0.21***		−0.20***
*F*	7.72***	11.85***	9.95***	9.46***	11.14***	18.01***	19.75***	14.40***	18.58***
*R* ^2^	0.08	0.14	0.16	0.09	0.13	0.23	0.28	0.24	0.29
Δ*R*^2^		0.06***	0.08***		0.04***	0.14***	0.18***	0.15***	0.20***

*N* = 371. *p < 0.05, **p < 0.01, ***p < 0.001.

**FIGURE 2:**
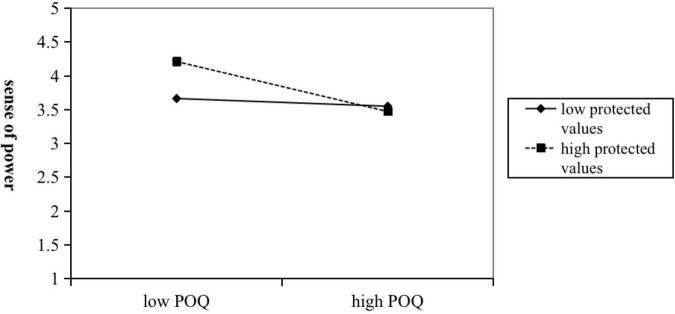
Two-way interaction effect between POQ and protected values on sense of power.

**FIGURE 3:**
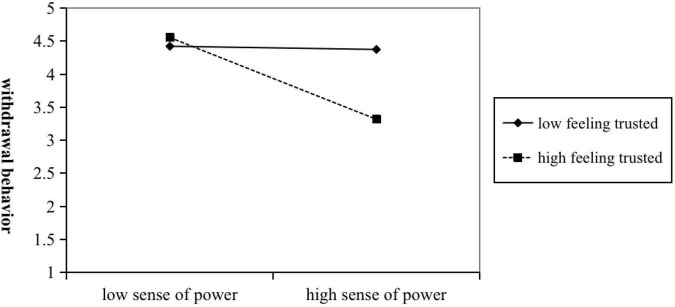
Two-way interaction effect between sense of power and feeling trusted on withdrawal behaviors.

Hypothesis 3a predicted that protected value moderates the effect of POQ on the sense of power, if the level of protective values is high, the negative relationship between POQ and the sense of power is strengthened. As shown in Table 3, the coefficient of the interaction terms of POQ and protected values to the individual sense of power was significant (M3: β = −0.10, *n.s.*). Especially, the results of the simple slope analysis in Figure 2, showed that if protected values were low, the effect between POQ and individual sense of power was not significant (β = −0.20, *n.s.*). However, if the coefficient of protected values was high, the effect between POQ and individual sense of power was significantly negative (β = −0.65, *p* < 0.001). Hypothesis 4a predicted that feeling trust Moderates the effect of the sense of power on the withdrawal behavior, if the level of feeling trust is high, the negative relationship between the sense of power and the withdrawal behavior is strengthened. As shown in Table 3, the coefficient of the interaction between the sense of power and feeling of trust in the withdrawal behavior was also significant (M7: β = −0.21, *p* < 0.001; M9: β = −0.20, *p* < 0.001). Additionally, the results of the simple slope analysis in Figure 3, showed that if the coefficient of the feeling of trust was low, the effect between the sense of power and the withdrawal behavior was not significant (β = −0.08, *n.s.*). But, if the coefficient of the feeling of trust was high, the effect between the sense of power and the withdrawal behavior was significantly negative (β = −0.50, *p* < 0.001).

To estimate two sets of effects at the high and low levels of the moderator, we employ the moderated path analysis approach (Edwards and Lamber, 2007) by Mplus. We analyzed the impact of POQ on the withdrawal behavior through the sense of power with different levels of protected values and feeling of trust, and the results are shown in Table 4. With diversified levels of protected values, the mediated effect of the sense of power varied significantly [Δβ = 2.42, *p* < 0.001, 95% CI = (0.01, 0.12)]. And, the mediated effect was strong [β = 3.14, *p* < 0.001, 95% CI = (0.01, 0.11)] as the level of protected values was high, vice versa [β = −0.10, *n.s.*, 95% CI = (−0.02, 0.02)]. Thus, Hypothesis 3b was supported. The mediated effect of the sense of power also varied significantly [Δβ = 2.86, *p* < 0.01, 95% CI = (0.02, 0.16)] within different levels of feeling of trust. Sequentially, the mediated effect was strong [β = 3.52, *p* < 0.001, 95% CI = (0.04, 0.19)] as the level of feeling of trust was high, vice versa [β = 1.33, *n.s.*, 95% CI = (−0.00,0.05)]. Thus, Hypothesis 4b was verified. We also test the two-stage moderated mediation using the path analysis approach in Table 5. The mediated effect of the sense of power was significant [Δβ = 3.09, *p* < 0.001, 95% CI = (0.03, 0.11)] only when both protected values and feeling trusted were high. In addition, all the hypotheses were verified without the four control variables.

**TABLE 4:** The results of moderated mediation.

		Indirect effect	95% CI
PV	+1 SD	3.14	[0.01, 0.11]
	−1 SD	−0.10	[−0.02, 0.02]
	Different effect	2.42	[0.01, 0.12]
FL	+1 SD	3.52	[0.04, 0.19]
	−1 SD	1.33	[−0.00, 0.05]
	Different effect	2.86	[0.02, 0.16]

**TABLE 5:** The results of two-stage moderated mediation.

		Indirect effect	95% CI
PV (+1 SD)	FL (+1 SD)	3.09	[0.03, 0.11]
	FL (−1 SD)	−0.27	[−0.04, 0.03]
PV (−1 SD)	FL (+1 SD)	−0.10	[−0.03, 0.03]
	FL (−1 SD)	0.03	[−0.01, 0.01]

## Discussion

There has been an expanding trend in researching the issue of POQ for high-level talents as an increased focus on high-level education employees. In this study, we developed the theory of power to discuss the mechanism and boundary conditions of POQ on employee withdrawal behavior. Specifically, we conducted the survey that objected to high-level education employees in three time periods separately and generated 371 valid questionnaires. In addition, we used hierarchical regression to test the hypothesis. First, we examine the mediation effect, the sense of power is a mediator between the effect of POQ on withdrawal behavior from the perspective of meeting the satisfaction of survival needs. Furthermore, we have considered how protected values, moderate the relationship between POQ and the sense of power. Also, we discussed how the feeling of being trusted by supervisors moderates the effect of the sense of power on the employees’ withdrawal behavior. Accordingly, we further explored the moderating effect of protected values and feeling trusted on the mediation of a sense of power between POQ and withdrawal behavior. Employees’ POQ would have a greater impact on withdrawal behaviors through a sense of power for those employees who have strong protected values and feel trusted by supervisors.

Our research, which is based on the power basis theory, explored the relationship between withdrawal behavior and POQ based on the satisfaction of survival needs. The results suggest that POQ can contribute to withdrawal behavior by enhancing the sense of power. The mediating effect of a sense of power can increase the impact of POQ on withdrawal behavior if workers have strong protected values or the feeling trusted. Our findings showed that POQ might affect the sense of power and lead to high withdrawal behaviors, particularly in those who have strong protected values and a higher level of trust in their superiors.

### Theoretical implications

The findings of this study have at least four theoretical implications. First, with the growing focus on the current knowledge economy and Talent Plan, POQ would like to make high-level talents a negative perception. Our primary contribution provides an advanced understanding of the relationship between POQ and employee withdrawal behavior, which captures the problem of high-level talents drain and provides new insights into the solution to preventing the drain. Considering risk type and prevention of the high-level talents drain, the studies discussed the risk triggered by market demand, legal risk, recruitment cost, government policy, and regulations (Chen et al., 2018), some previous studies analyzed the preventive measures from the perspective of regime, policy, capital, and mechanism (Chen et al., 2018). Although few studies discussed withdrawal behavior from the perspective of self-perception factors, it is the core research theme in our article, our article provides a new perspective.

Second, we contribute to a growing line of research on POQ and anti-production behavior. Thus far, the studies on the effect of POQ mainly focused on active behavior (Harari et al., 2017). What’s more, a variety of previous research just analyzed the relationship between the certain manifestation of withdrawal behavior and POQ. Harari et al. (2017), for instance, explored the relationship between POQ and departure willingness and behavior. According to Chen et al. (2020), the humble quality of leadership could accommodate the positive relationship between POQ and departure willingness. Maynard and Parfyonova (2013) showed the mediating effect of job satisfaction and emotional commitment on the relationship between POQ and active job search behavior, which can be moderated by competency and growth values. However, utilizing a three-phase methodology, we directly examined withdrawal behavior to deeply understand the relationship between POQ and withdrawal behaviors.

Third, the basic theory of power served as the foundation for our research, which started from the necessity for survival. This theory helped to explain why a lack of use of individual unique talents would lead to a loss in power and the transfer of high-level talent to another entrepreneur. The employees’ POQ prevents them from gaining the power they desire, which in turn prevents them from meeting their survival needs. Hence, high-level talents choose to switch to others for meeting their survival needs triggering amounts of withdrawal behavior. The research on the effect mechanism of POQ is discussed from the perspective of social exchange, resources, and equity (e.g., Fine and Edward, 2017; Chen et al., 2020). Taken as a whole, our findings suggest that the sense of power mediates the impact of POQ on withdrawal behavior, which provides new insights into the internal mechanism of relationships and enriches the basic theory of power.

Finally, we discover that protected values and a sense of trust have a moderating effect on the path of POQ—the sense of power—withdrawal behavior. Previous studies verified that employees respond differently to POQ (Liu et al., 2015; Arvan et al., 2019; Woo, 2020). The study discussed the boundary condition of POQ with protected values since the protected values of high talents are stronger than those of ordinary individuals. In addition, our research explored the moderating effect of feeling trusted from a leadership perspective, which enriches the mechanism of perceived leadership trust and adds to the boundary conditions.

### Practical implications

Along with these conceptual advantages, our research has significant practical implications. First, the POQ would cause significant losses in both economy and human resources. Also, the Talent Plan’s effectiveness will be negatively impacted by the brain drain brought on by employees’ withdrawal behaviors. Our findings thus proposed several rectification measures and practical implications for solving the problem of POQ and withdrawal behavior. First, the human resources planning should be modified to prevent blindly pursuing high-level talents, which results in POQ and an unbalanced workforce allocation. Furthermore, to evaluate the skills, knowledge, and ability of high-level talents, the organization needs to attach the importance of creating favorable working conditions.

Second, we suggest entrepreneurs take the sense of power into account for preventing POQ and withdrawal behavior. In other words, the negative effect of POQ can be solved by improving their sense of power. For example, we could make high-level talents get a promotion, provide more convenience and work support, and even assign other employees to cooperate with them to finish arrangements. In this way, it can increase the sense of power to lessen withdrawing behavior while simultaneously encouraging high-level talents to sufficiently utilize their skills to improve the fulfillment of survival needs. Additionally, the organization needs to express the cultural concept of “respecting knowledge and talents” in order to strengthen the status of high-level talents in the organization and help them develop their psychological bond with the organization and adopt more proactive behaviors.

Finally, our findings suggest emphasizing protected values and a feeling of trust for enhancing the intermediary role of an individual’s sense of power. This makes recruitment not only concerned with the ability but also with evaluating their valuation. In another way, to reduce the withdrawal behavior brought on by POQ, the organization should act quickly if the employees have highly protected values, for example, by raising job demands or switching the guard. Meanwhile, the organizations also need to allocate the extent of trust. Leadership trust can help to improve employee withdrawal behavior because individuals feel more empowered. But when there is a low sense of power, the feeling of trust will have a negative effect.

### Limitations and future research directions

This research should be considered with several limitations that offer important avenues for promising future research. First, even though we used a three-period questionnaire collection approach, the interference of homologous data could not be eliminated because only one employee filled out all the variables, some of which were filled out simultaneously. In further research, to address the problem of common method bias, we would like to use the leader-employee matching approach.

Secondly, we assumed that objective conditions were to blame for the sensation of overqualification. However, the employees would like to conduct overqualified for cost more on their families, fitness, and learning new skills and knowledge (Pratto et al., 2011). Due to this, the POQ can only partially satisfy some survival needs, while other needs, such as those for affection, good physical and mental health, and social engagement, all can be met. In other words, the impact of the POQ on the individual’s sense of power is limited, down to no impact. Consequently, we will explore how POQ affects individual cognition and behavior in the way of objection and subjection, respectively. Future research will also examine the impact of various marginal conditions on how well individual needs to alter the POQ effect degree are met.

Finally, although protected values and a sense of trust were taken into account in our research, the crucial influential component, colleagues, was not. Employees are highly affected by the treatment of their colleagues due to the comparison with others for judging fairness, this results in changing perception of POQ caused by the case that their colleagues are in POQ. Based on the theory of fairness heuristic, it was demonstrated that when the organization had a high level of interactive fairness, POQ could produce extremely positive results (Zhou et al., 2020). Therefore, we urge future researchers to consider the fairness factors in the research of POQ.

## Data availability statement

The raw data supporting the conclusions of this article will be made available by the authors, without undue reservation.

## Ethics statement

The studies involving human participants were reviewed and approved by the IRB of College of International Economics and Trade, Ningbo University of Finance and Economics. The patients/participants provided their written informed consent to participate in this study.

## Author contributions

CH conceived the study, designed questionnaires, wrote the article in Chinese, and finalized the manuscript for submission. ST and RW collected data. XW was responsible for draft writing in English and literature collation. All authors contributed to the article and approved the submitted version.
